# Left Atrial Wall Thickness Estimated by Cardiac CT: Implications for Catheter Ablation of Atrial Fibrillation

**DOI:** 10.3390/jcm13185379

**Published:** 2024-09-11

**Authors:** Pedro Silva Cunha, Sérgio Laranjo, Sofia Monteiro, Inês Grácio Almeida, Tiago Mendonça, Iládia Fontes, Rui Cruz Ferreira, Ana G. Almeida, Maxim Didenko, Mário Martins Oliveira

**Affiliations:** 1 Cardiology Service, Arrhythmology, Pacing and Electrophysiology Unit, Hospital Santa Marta, 1169-024 Lisbon, Portugal; sergio.laranjo@ulssjose.min-saude.pt (S.L.);; 2 Instituto de Fisiologia, Faculdade de Medicina, University of Lisbon, 1649-004 Lisbon, Portugal; 3 Comprehensive Health Research Center, NOVA Medical School, Faculdade de Ciências Médicas, NMS, FCM, Universidade NOVA de Lisboa, 1169-056 Lisbon, Portugal; 4 Departamento de Fisiologia, NOVA Medical School, Faculdade de Ciências Médicas, NMS, FCM, Universidade NOVA de Lisboa, 1099-085 Lisbon, Portugal; 5 Instituto de Telecomunicações, Instituto Superior Técnico, 3810-193 Aveiro, Portugal; 6 Imagiology Department, Hospital Santa Marta, 1169-024 Lisbon, Portugal; 7 CCUL@RISE, Faculdade de Medicina, Universidade de Lisboa, 1649-028 Lisbon, Portugal; 8 Heart and Diabetes Center NRW, University Clinic of the Ruhr University Bochum, 44789 Bochum, Germany

**Keywords:** atrial fibrillation, atrial wall thickness, ganglionated plexi, catheter ablation, left atrium, multi-detector computed tomography, atrial remodelling, substrate, recurrence-free survival, personalised treatment

## Abstract

Atrial wall thickness (AWT) is a significant factor in understanding the pathological physiological substrate of atrial fibrillation, with a potentially substantial impact on the outcomes of catheter ablation procedures. Precise measurements of the AWT may provide valuable insights for categorising patients with AF and planning targeted interventions. **Objectives:** The purpose of this study was to evaluate the characteristics of the left atrium (LA) using non-invasive multidetector computed tomography (MDCT) scans and subsequent three-dimensional (3D) image post-processing using novel software designed to calculate atrial thickness dimensions and mass. **Methods:** We retrospectively analysed 128 consecutive patients (33.6% females; mean age 55.6 ± 11.2 years) referred for AF ablation (37 with persistent AF and 91 with paroxysmal AF) who underwent preprocedural MDCT. The images were post-processed and analysed using the ADAS software (Galgo Medical), automatically calculating the LA volume and regional wall thickness. In addition, the software employed a regional semi-automatic LA parcellation feature that divided the atrial wall into 12 segments, generating atrial wall thickness (AWT) maps per segment for each patient. **Results:** This study demonstrated considerable variability in the average thickness of LA walls, with the anterior segments being the thickest across the cohort. Distinct sex-specific differences were observed, with males exhibiting greater anterior and septal wall thickness than females. No significant associations were identified between the average AWT and body mass index, LA volume, or sphericity. Survival analysis conducted over 24 months revealed a meaningful relationship between mean anterior wall thickness and recurrence-free survival, with increased thickness associated with a lower likelihood of AF-free survival. No such relationship was observed for the indexed LA volume. **Conclusions:** The variability in AWT and its association with recurrence-free survival following AF ablation suggest that AWT should be considered when stratifying patients for AF management and ablation strategies. These findings underscore the need for personalised treatment approaches and further research on the interplay of the structural properties of the left atrium as factors that can serve as important prognostic markers in AF treatment.

## 1. Introduction

Atrial fibrillation (AF) is the most prevalent arrhythmia encountered in clinical practice, affecting millions of people worldwide [1]. While the pathophysiology of AF is multifaceted, there is growing acknowledgement of the pivotal role played by the structural remodelling of atrial tissue [2].

The left atrium (LA) is critical for maintaining effective cardiac function, serving as a reservoir, conduit, and booster pump during the cardiac cycle [3]. It aids blood flow from the pulmonary veins into the left ventricle, significantly influencing cardiac output and haemodynamics. Consequently, alterations in the LA structure, such as changes in wall thickness, can profoundly affect atrial function and predispose individuals to various cardiovascular conditions.

Atrial wall thickness (AWT), atrial morphology, the extent of histological changes, and the autonomic nervous system have emerged as critical determinants in understanding the pathophysiology of AF [4]. Various studies have demonstrated that increased AWT is associated with heightened fibrosis, altered conduction properties, and increased susceptibility to the initiation and maintenance of AF [5,6]. Moreover, atrial morphology and chamber size changes, particularly during a cardiac autonomic nervous system imbalance, have also been linked to AF susceptibility and progression [7,8,9]. Additionally, histological changes such as myocyte hypertrophy, inflammation, fibrosis, and fatty infiltration are crucial for the creation of a substrate for AF initiation and perpetuation [2,10]. Among these structural characteristics, the AWT [11] has emerged as a promising parameter with essential implications for understanding the underlying mechanisms of AF.

Accurately evaluating the left AWT has historically posed challenges, often requiring invasive procedures or relying on limited imaging modalities. When examined ex vivo, tissue manipulation and formalin fixation introduce potential sources of error when characterising the anatomical structure of the left atrium within thoracic tissue samples [11]. However, in recent years, advancements in imaging modalities have offered non-invasive means to assess AWT, providing clinicians with valuable insights into the disease process [12]. Contrast-enhanced cardiac computed tomography (CE-CT) has emerged as a potent diagnostic tool that delivers detailed anatomical information and functional insights regarding heart anatomy [11]. Among the various parameters that can be assessed using CE-CT, the measurement of the left AWT has gained particular importance because of its implications for AF ablation [13]. Understanding the left AWT can provide valuable insights into the pathophysiology of AF, aid in identifying patients at a higher risk of complications, and assist in planning optimal treatment strategies [14,15]. Our study aims to identify new indicators and predictors of post-ablation success, contributing to the ongoing search for markers that can improve patient outcomes.

## 2. Materials and Methods

### 2.1. Study Population and Design

This study was a single-centre retrospective observational analysis of patients who underwent elective AF ablation at our tertiary care facility between January 2019 and January 2022. The “Centro Hospitalar de Lisboa Central” ethics committee approved the study protocol (Ethics Committee approval number 974/2020), and all participants provided written informed consent for data collection. This study adhered to the principles outlined in the Declaration of Helsinki.

The AF documentation was based on electrocardiograms or Holter monitoring before radiofrequency catheter ablation (RFCA).

The primary objective of this study was to examine the association between atrial thickness and mass parameters in patients with AF, focusing on their potential as early indicators of disease progression. Additionally, as a secondary aim, we sought to investigate whether any observed parameters correlated with arrhythmia recurrence during the follow-up period.

The study cohort consisted of consecutive adult patients aged ≥18 years who presented with symptomatic paroxysmal or persistent AF and were referred for catheter ablation as a part of their clinical care. The inclusion criterion was the performance of a CE-CT scan within two days before ablation. Treatment-naïve patients and those previously managed with pharmacological therapy for AF, including rate control, rhythm control, and anticoagulation medications, were included. Patients who underwent prior AF ablation or AF-related surgery were excluded from the study.

One hundred and twenty-eight patients met the inclusion criteria and were included in this study. Baseline demographic, clinical, and echocardiographic data were extracted from electronic medical records, including information such as age, sex, body mass index, AF subtype (paroxysmal or persistent), AF duration, comorbidities, prescribed medications, LA diameter, and left ventricular ejection fraction.

### 2.2. Contrast-Enhanced Multi-Detector Computed Tomography

Patients underwent preprocedural perfusion multidetector computed tomography (MDCT) using a 128-slice dual-source cardiac scanner with electrocardiographic gating and iodinated contrast product administration. Local standardised practices were used to conduct acquisition protocols, interpretation, and reporting.

The CE-CT scan was triggered when attenuation of the region of interest (ROI) in the ascending aorta reached 100 Hounsfield units (HU) for 10 s. The contrast agent, Visipaque™ (iodixanol, 320 mg), was injected through a peripheral vein at 5 mL/s, followed by a 25 mL saline bolus chase. The scan parameters included a collimation of 0.625 mm, rotation time of 350 ms pitch adjusted to each patient’s heart rate, tube voltage of 80–120 mV, and effective mA of 100–600.

The images were preprocessed to enhance their quality and suitability for further analysis. This includes noise reduction, standardisation of pixel values, and artefact correction.

### 2.3. Image Post-Processing

**Figure 1:**
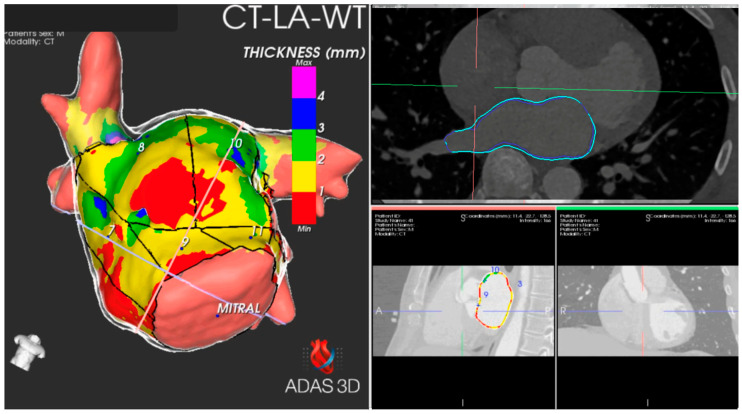
Software reconstruction using multidetector computed tomography images and analysis using ADAS 3D™ software generated a 3D map of the atrial wall thickness. Thickness colour map: thickness <1 mm, red; 1–2 mm, yellow; 2–3 mm, green; 3–4 mm, blue; >4 mm, purple.

The CE-CT data acquired before the procedure were exported in a compatible format to facilitate further analysis using the artificial intelligence-based automatic detection of arrhythmic substrate (ADAS) software (version 2.12.1) (Galgo Medical SL, Barcelona, Spain). The ADAS 3D™ software was used to study MDCT images, allowing for the generation of a three-dimensional map representing the AWT. The AWT calculation was conducted in two stages. Initially, the software delineated the endocardial layer using pixel intensity thresholds and then segmented the endocardial and epicardial layers. Subsequently, manual delineation of the pulmonary veins, left atrial appendage, and mitral area was performed. To determine wall thickness, the artificial intelligence software computed the distance between each endocardial point and its corresponding projection onto the epicardial shell (Figure 1). This process resulted in a three-dimensional wall thickness map, which was subsequently divided into 1 mm intervals to facilitate operator-guided selection. On average, the image processing task was completed by a skilled operator within approximately 12 ± 2 min.

**Figure 2:**
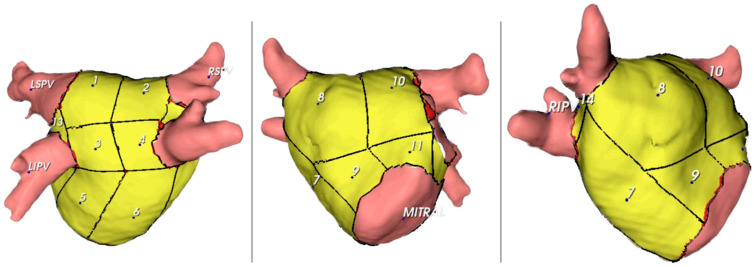
Schematic illustration of 14 locations where the wall thickness was measured in the left atrium. Posterior (**left panel**), anterior (**middle panel**), and left lateral views (**right panel**) are presented. Legend: Segments 1–4, superior wall; 5–6, posterior wall; 7, septal wall; 8–11, anterior wall; 12, left lateral wall; and 13–14, between superior and inferior pulmonary veins. LIPV, left inferior pulmonary vein; LSPV, left superior pulmonary vein; MITRAL, mitral annulus; RIPV, right inferior pulmonary vein; RSPV, right superior pulmonary vein.

Each patient was assigned a unique identifier, and the left AWT was measured in 14 distinct segments. The 14-segment model (as illustrated in Figure 2) anatomically corresponds to the following areas: segments 1–4 represent the superior wall; segments 5–6 pertain to the posterior wall; segment seven is associated with the septal wall; segments 8–11 relate to the anterior wall; segment 12 covers the left lateral wall; and segments 13–14 are located between the superior and inferior homolateral pulmonary veins. These measurements included the mean thickness and standard deviation of the thickness of each segment, along with the total mass. The total mass was measured in the left inferior pulmonary vein (LIPV), left superior pulmonary vein (LSPV), right inferior pulmonary vein (RIPV), right superior pulmonary vein (RSPV), and left atrial appendage (LAA). Additionally, we computed LA volume, LA body volume, and sphericity index for the LA body.

### 2.4. Ablation Procedure

Our approach has been previously described [16]. Before the ablation procedure, each patient underwent a routine preprocedural transthoracic echocardiogram to assess the left ventricular ejection fraction and LA dimensions, as well as computed tomography (with segmentation of the LA) to evaluate the left atrial anatomy and exclude the presence of intracardiac thrombi. Patients were administered oral anticoagulants using warfarin with a therapeutic INR (2.0–3.0) or direct oral anticoagulants (DOACs), with one dosage omitted before ablation. All antiarrhythmic drugs (AAD) were discontinued for at least five half-lives before the procedure. Continuous oxygen saturation and ECG monitoring were maintained throughout the ablation process and performed under conscious sedation.

In summary, the protocol for RFCA involved the following steps: (1) positioning a decapolar catheter through the right femoral vein to the coronary sinus to guide the transseptal puncture and pace the LA, (2) performing a transseptal puncture via fluoroscopic guidance, and (3) utilising the CARTO system (Biosense Webster, Irvine, CA, USA) for three-dimensional mapping of the LA. Radiofrequency applications were performed using an open irrigated-tip catheter (ThermoCool SmartTouch^®^ SurroundFlow; Biosense Webster) with point-by-point lesions. In all patients, the ablation strategy involved antral isolation, aiming at a contiguous circle enclosing the pulmonary veins without additional lines. A real-time automated display of the RF applications (Visitag, Biosense Webster) was used with predefined settings of catheter stability (3 mm for 8 s) and minimum CF (30% of time >4 *g*). RF was delivered (EP Shuttle ST-3077, Stockert, Freiburg, Germany) in power-controlled mode at 25 to 35 W (irrigation flow up to 30 cc/min). RF was delivered until an AI of ≥400 at the posterior wall/roof and ≥500 at the anterior wall. A new RF application reaching the AI target was applied in case of dislocation. The maximal interval between 2 neighbouring lesions was ≤6 mm [17].

### 2.5. Post-Ablation Evaluation

The principal objective of this study was to evaluate the recurrence-free survival (RFS) of patients with AF, defined as the absence of any episodes of atrial tachyarrhythmia lasting at least 30 s beyond a 90-day blanking period. These included sustained symptomatic episodes of rapid palpitations.

Following the ablation procedure, the patients were discharged using AAD and oral anticoagulants, which were determined at the operator’s discretion. Regular follow-up appointments were scheduled at the outpatient clinic, 1–3 months post-procedure, and subsequently every six months (or sooner if symptoms arose) within the initial two years post-ablation. Upon completing the blanking period, patients underwent 24-h Holter monitoring during each outpatient visit, and a standard 12-lead electrocardiogram (ECG) was performed during each visit. Antiarrhythmic medication was discontinued three to six months after the ablation procedure if the patient remained symptom-free regarding arrhythmia. In the third month, oral anticoagulation was re-evaluated based on the CHA2DS2-VASc score, with the decision to continue contingent on this assessment. Throughout the follow-up period, the clinical events were thoroughly assessed and recorded.

### 2.6. Statistical Analysis

A statistical analysis examined the relationships between the thickness and mass of left atrial (LA) segments and walls and various patient characteristics. The variables analysed included atrial fibrillation (AF) type, sex, AF recurrence status, redo procedure status, body mass index (BMI) categories, number of comorbidities, LA body volume, LA sphericity, and individual comorbidities.

Depending on their distribution, continuous variables were described using either mean ± standard deviation (SD) or median with interquartile range (IQR). Categorical variables are presented as frequencies and percentages. The normality of continuous variables was assessed using the Shapiro–Wilk test, supplemented by visual inspection of histograms and QQ plots.

The initial sample analysis involved creating frequency tables for categorical variables and computing descriptive statistics for continuous variables, specifically means and standard deviations. It also included calculating the thickness and mass of each left atrial (LA) segment and wall.

Correlation analyses were performed to investigate the associations of the thickness and mass of the LA segments and walls with various factors. These factors included the type of atrial fibrillation (AF), sex, AF recurrence status, redo procedure status, body mass index (BMI), number of comorbidities, LA body volume, and LA sphericity. BMI was categorised based on the World Health Organization criteria, and the ‘maximally selected rank statistics’ method was employed to determine the optimal cut-off for the number of comorbidities.

Chi-square tests were used to analyse categorical variables, whereas a Pearson’s correlation coefficient was applied to continuous variables. The Bonferroni correction was used to adjust for multiple testing, with the significance level set at *p* < 0.05.

Survival analysis was conducted to estimate arrhythmia recurrence-free survival concerning left atrial characteristics. Kaplan–Meier survival curves were generated, and the log-rank test was used to compare survival distributions. The ‘cutpoint’ package in R was utilised to identify optimal cut-off points for critical variables such as LA volume index and anterior wall thickness, aiming to maximise the sum of sensitivity and specificity for survival outcomes.

All statistical analyses were performed using R version 4.1.2 and RStudio version 1.4.1106.

## 3. Results

### 3.1. Patient Characteristics

The study cohort was comprised of 128 individuals who were referred for AF ablation. The participants ranged in age from 24 to 78, with a mean age of 55.6 ± 11.2 years. Most participants (66.4%) were male, with a male-to-female ratio of approximately 2:1, and 71% had paroxysmal AF. During the 24-month follow-up, AF recurrence was observed in 43% of the cases, and a repeat procedure was required in 10.94%. A summary of the baseline characteristics of the groups is shown in Table 1.

A substantial proportion of patients were classified as overweight (42.97%), with normal weight being the next most prevalent category (29.69%) and obesity accounting for 27.34% of the sample. None of the patients belonged to the underweight category.

The distribution of CHA2DS2-VASc scores varied considerably among patients. The largest group scored 1 (37.50%), followed by 2 (25.00%), 0 (20.31%), and 3 (14.06%). Only a small number of patients had scores of four (2.34%) or five (0.78%).

The study population displayed a diverse range of comorbidities, with arterial hypertension being the most prevalent, affecting 61.72% of patients. Hyperlipidemia was also present in 27.34% of the patients. Simultaneously, diabetes mellitus, obstructive sleep apnoea syndrome, smoking, chronic obstructive pulmonary disease, chronic kidney disease, heart failure, coronary disease, vascular disease, previous stroke, thromboembolism, and hypertrophic cardiomyopathy were found in a small number of patients.

### 3.2. Descriptive Analysis

#### 3.2.1. Thickness of Segments and Walls

The present study aimed to examine the thickness of various segments and walls of the LA and identify patterns across these regions. The thicknesses of 14 distinct segments of the LA walls were measured in our analysis, as depicted in Figure 2. After excluding pulmonary veins, LA appendage, and mitral valve, 14 segments were established in the atrial wall: segments 1–4, superior wall; 5–6, posterior wall; 7, septal wall; 8–11, anterior wall; 12, left lateral wall; and 13–14, between superior and inferior homolateral pulmonary veins.

**Figure 3:**
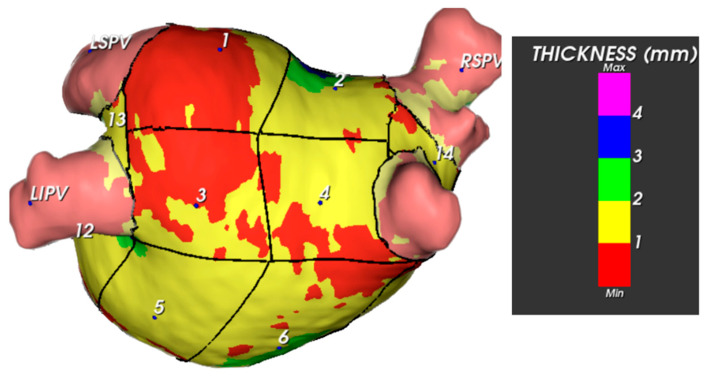
Example of LA wall thickness measurement (posterior view). The bar on the right shows the colour code assigned to the thickness variations.

The thicknesses of these segments varied considerably (Figure 3). Specifically, segments 8 and 10 (the anterior wall close to the right superior pulmonary veins and the anterior wall near the left atrial appendage ostium and left superior pulmonary vein, respectively) were noted to be the thickest on average. In males, segment 8 exhibited a mean thickness of 1.98 ± 0.99 mm; in females, this segment displayed a mean thickness of 1.73 ± 0.56 mm (Table 2). Additionally, in males, segment 10 had a mean thickness of 2.22 ± 1.13 mm, whereas in females, it had a mean thickness of 1.97 ± 0.70 mm. Segment 9 displayed the lowest thickness, with a mean value of 1.28 ± 0.27 mm.

Anatomically, the anterior wall was the thickest on average, with a mean thickness of 1.66 ± 0.29 mm. In contrast, the septum had the smallest thickness among the walls, with a mean thickness of 1.32 ± 0.23 mm.

The diverse thickness values across different segments and walls reflect the heterogeneous nature of the left atrial wall in patients with AF.

#### 3.2.2. Mass of Segments and Walls

Our examination of the mass of the segments and walls uncovered another layer of variability within the left atrial components. Among the 14 segments, segment 8 (the anterior wall near the right superior pulmonary vein) displayed the highest average mass, with a mean of 2.68 ± 0.92 g. Conversely, segment 3 (the posterior wall near the left inferior pulmonary vein) had the lowest average mass, with a mean of 0.69 ± 0.27 g.

The posterior wall displayed the highest average mass, with a mean of 1.69 ± 0.58 g. In contrast, the region between the pulmonary veins had the lowest average mass, with a mean of 0.38 ± 0.18 g.

#### 3.2.3. Data Analysis

**Figure 4:**
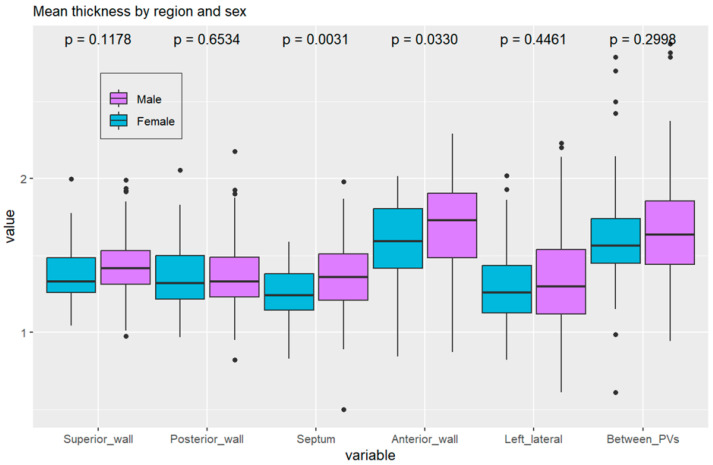
Boxplot of left atrial wall thickness distribution according to region.

Significant correlations were identified between the thickness and mass of LA wall segments and patient characteristics. Differences in thickness between sexes were statistically significant in the anterior and septal regions, with males showing higher values than females (Figure 4). These differences may influence ablation strategies, as power and application time should be selected considering the lower thickness values in females.

Figure 4 shows the thickness of the walls by anatomical region and sex, indicating a statistically significant difference between sexes in the septal and anterior walls (*p* = 0.03 for the anterior wall, *p* = 0.003 for the septal wall).

A significant correlation was also found between the thickness of the left inferior pulmonary vein (LIPV) and the type of AF, with a *p*-value of 0.02. This suggests a possible association between LIPV thickness and AF type, although the direction and magnitude of this relationship should be interpreted with caution.

**Figure 5:**
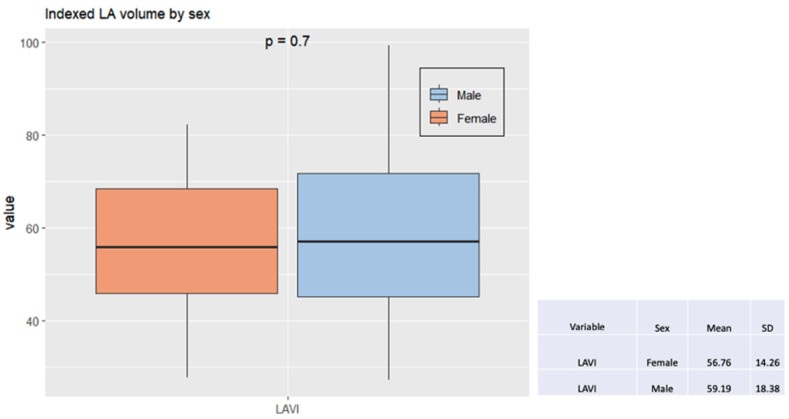
Box plot with comparison by sex of the left atrial volume index to the body surface. Legend: LAVI, left atrial volume index; SD, standard deviation.

The analysis indicated that males and females had similar left atrial volume index (Figure 5).

**Figure 6:**
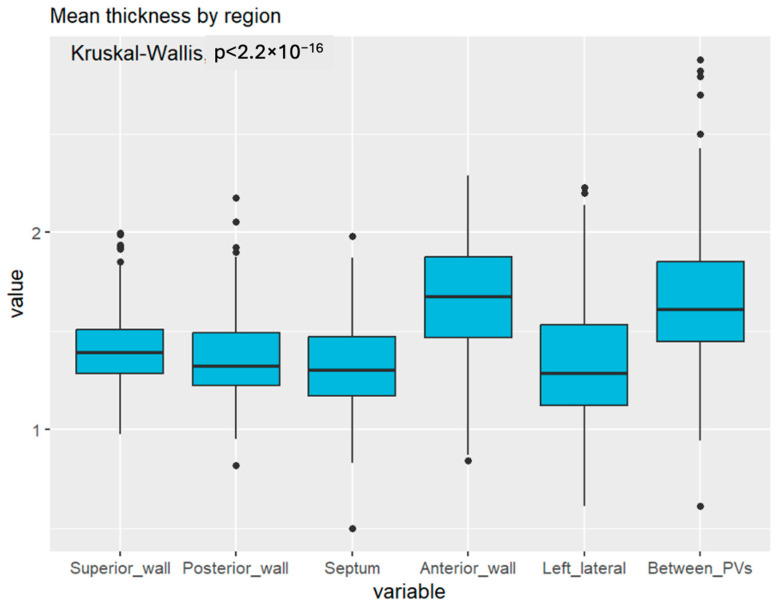
Mean left atrial wall thickness by region.

**Figure 7:**
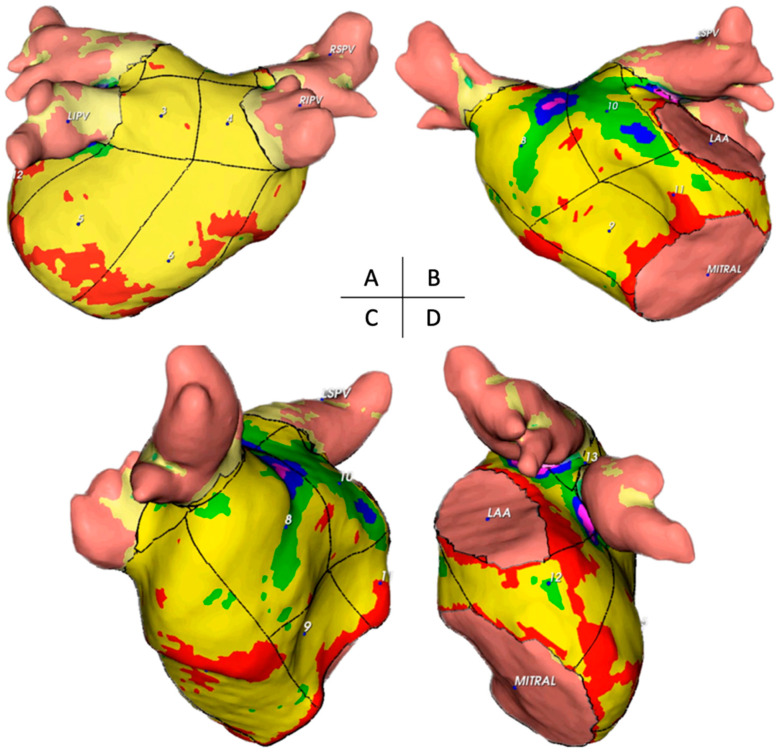
Illustration of the 14 locations in the left atrium and corresponding topographic colour maps showing thickness differences. Posterior and anterior views (**A**,**B**), right anterior oblique view (**C**), and left lateral view (**D**) are shown. Legend: LIPV, left inferior pulmonary vein; RIPV, right inferior pulmonary vein; RSPV, right superior pulmonary vein; MITRAL, mitral annulus; LAA, left atrial appendage ostium.

From the point of view of anatomical description, it is essential to recognise that in the LA, the thickest regions correspond to the anterior wall and the area between the pulmonary veins (Figure 6 and Figure 7 and Table 2).

#### 3.2.4. Correlations with BMI, Left Atrial Characteristics, and Comorbidities

Our investigation was extended to examine correlations between the thickness and mass of the segments and walls and various factors, including BMI categories, left atrial body volume, sphericity, and individual comorbidities. We found no statistically significant correlations.

### 3.3. Survival Free of AF Analysis

#### 3.3.1. Anterior Wall Thickness

**Figure 8:**
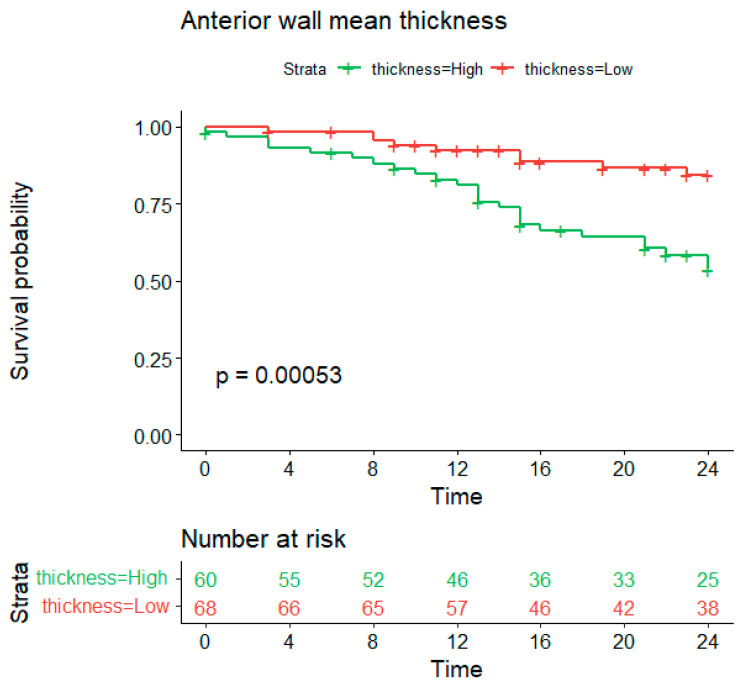
Log-rank test estimates of recurrence-free survival according to mean anterior wall thickness (optimal cutpoint → 1.69 mm).

The relationship between mean anterior wall thickness and recurrence-free survival over a 24-month follow-up period was analysed using the Kaplan–Meier method. Patients were divided into ‘high’ (>1.69 mm) and ‘low’ (≤1.69 mm) thickness groups based on an optimal cut point of 1.69 mm. The log-rank test indicated a statistically significant difference in survival probabilities between the two groups (*p* = 0.00053), with the high-thickness group demonstrating a lower survival probability throughout the study duration (Figure 8). Discriminant analysis identified the thickness of the anterior wall (optimal cut-off point of 1.69 mm, *p* = 0.00053) and LAVi as the strongest predictors of arrhythmic recurrence. At the start of the follow-up, the number of patients at risk in the ‘high’ thickness group was 60, and in the ‘low’ thickness group, it was 68. By the end of the study, these numbers had decreased to 25 and 38, respectively.

#### 3.3.2. Indexed Left Atrial Volume

**Figure 9:**
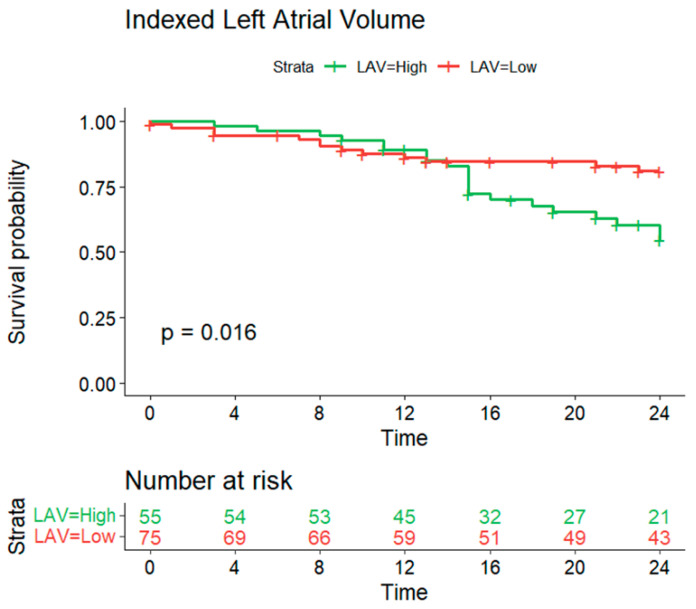
Survival curve estimates of arrhythmia recurrence-free survival according to left atrial index volume. Legend: The optimal cut-off point obtained by maximising the sum of sensitivity and specificity was 58.6 mL/m^2^.

The Kaplan–Meier method was also used to evaluate the impact of the indexed left atrial volume (LAV) on recurrence-free survival over a 12-month follow-up period. Patients were stratified into ‘high’ (>51.163) and ‘low’ (≤51.163) LAV groups. The log-rank test revealed a statistically significant difference in survival probability between these groups (*p* = 0.016) (Figure 9). Initially, there were 79 patients in the ‘high’ LAV group and 51 in the ‘low’ LAV group. By the end of the study, the number of at-risk patients had decreased to 68 and 36, respectively.

## 4. Discussion

The primary objective of this study was to investigate the variations in wall thickness (WT) in patients undergoing atrial fibrillation (AF) ablation. Our analysis included a diverse cohort of patients with various comorbidities and demographic characteristics. Using angio-CT scans and artificial intelligence ADAS software from Galgo^®^, we characterised the left WT and mass in 14 segments by reconstructing both the epicardial and endocardial surfaces and generating wall-thickness maps. This study advances our understanding of left WT, an area with limited in vivo data.

### 4.1. Main Findings

**Figure 10:**
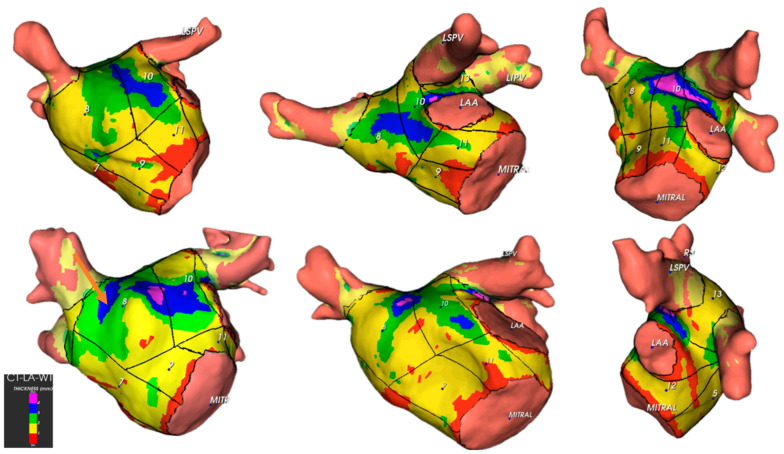
Illustration of left anterior wall thickness in different patients. The areas with the highest thickness corresponded to the Bachmann bundle region. Legend: LIPV = left inferior pulmonary vein, LSPV = left superior pulmonary vein, RIPV = right inferior pulmonary vein, RSPV = right superior pulmonary vein, MITRAL = mitral annulus, LAA = left atrial appendage ostium.

Our investigation revealed significant disparities in wall thickness across various atrial segments. The anterior segments were the thickest (Figure 10), which is relevant for catheter ablation strategies, mainly when targeting cardiac neuromodulation of atrial ganglionated plexi. This finding was consistent for both men and women. Notably, men exhibited thicker anterior and septal walls than women, suggesting the need for adjustments in ablation approaches, such as power settings and application time, to optimise outcomes and minimise complications.

The anatomical composition of the anterior wall includes the Bachmann bundle and deeper portions of the septopulmonary bundle adjacent to the anterior segment of the right superior pulmonary vein, along with autonomic ganglia conglomerations [18]. These regions are critical to the heart’s electrophysiological properties. Increased thickness at these locations may affect the efficacy and safety of ablation therapy because of their proximity to critical conduction pathways.

Beyond the wall thickness, the study also analysed the mass of the left atrial segments, revealing similar variability. The anterior wall near the right superior pulmonary vein had the highest average mass, indicating significant structural remodelling in response to AF. These findings suggest that the mass in the left atrial segments is an essential factor in managing arrhythmias and should be considered in therapeutic strategies.

The study also explored the relationships between structural attributes and patient characteristics and found significant correlations between AWT, sex, and AF type. However, after adjusting for multiple comparisons, no significant correlations were observed between BMI, left atrial body volume, sphericity, or comorbidities. This complexity highlights the potential influence of unmeasured factors.

#### 4.1.1. Left Atrial Volume Index, Atrial Wall Thickness, and AF Recurrence

Survival analysis for over 24 months provided valuable prognostic information. Owing to the irregular shape of the LA, the left atrial volume (LAV) is a more accurate measure of size than diameter. A meta-analysis found that patients with AF recurrence after radiofrequency ablation had a higher mean left atrial volume indexed to body surface area (LAVi) compared to those without recurrence, and this was independently associated with an increased risk of AF recurrence [19].

This study found a small difference between patients with and without AF recurrence regarding LAVi (0.6 mL/m^2^, CI-0.305–0.888). An optimal cutoff point of 58.6 mL/m^2^ was identified for predicting recurrence probability. The precise relationship between left atrial enlargement and AF remains unclear, but larger LA size is suggested to perpetuate atrial remodelling and AF [20,21,22].

A strong association was found between mean anterior wall thickness and recurrence-free survival, with increased thickness correlating with lower survival probability. This highlights the potential of anterior wall thickness as a prognostic marker of patient outcomes following AF ablation.

#### 4.1.2. Mechanisms of Recurrence

Discriminant analysis identified the thickness of the anterior wall (optimal cut-off point of 1.69 mm, *p* = 0.00053) and LAVi as the strongest predictors of arrhythmic recurrence. Pulmonary vein reconnection is primarily caused by non-transmural ablation [23,24]. Recent studies have shown a correlation between local atrial wall thickness and acute pulmonary vein reconnection following pulmonary vein isolation (PVI) [25]. Acute reconnection was observed in segments with greater local AWT in the anterior/roof and posterior/inferior regions. Another study reported a higher AWT in reconnected segments of the pulmonary veins [26]. Although this study did not evaluate early or late reconnection, increased anterior wall thickness may have contributed to higher recurrence rates.

Several studies have indicated that the atrial wall has a bilayer structure with fibres in the epicardial and endocardial layers oriented in nearly perpendicular directions [26,27,28]. Using three-dimensional diffusion tensor magnetic resonance imaging, researchers have observed a bilaminar muscular structure in certain atrial regions, whereas fibre angles in the other areas remained relatively consistent [29].

### 4.2. Clinical Implications

This study identified anterior wall thickness as a significant anatomical predictor of AF recurrence following single PVI-based AF ablation. The LA cavity index dimensions were also associated with arrhythmia recurrence at the 24-month follow-up. These parameters can be determined using preprocedural 3D-CT imaging. Recognising the AWT is valuable for patient selection and ablation strategy planning, including power settings and regions for ganglionated plexus ablation [30,31,32].

Future research should aim to gather data on atrial wall changes and remodelling due to AF progression, developing an AWT atlas for patients with AF. Integrating these data with three-dimensional mapping systems can combine electrical and structural information, enhance the understanding of AF pathophysiology, and improve ablation treatment strategies.

### 4.3. Limitations

With a limited sample size, this single-centre study may be susceptible to selection bias and reduced generalisability. Manual segmentation and analysis using specific software restricted the number of patients analysed. The study was limited to patients undergoing PVI alone and considered only the left atrial body, excluding the left atrial appendage, pulmonary vein insertions, and the right atrium. Future investigations, including longitudinal and mechanistic studies, are necessary to determine the causative relationships and to understand the underlying mechanisms.

Despite these constraints, our findings provide relevant anatomical details for future clinical practice and warrant further exploration to generate further hypotheses. A Bonferroni correction was applied to adjust the *p*-values for multiple comparisons, reducing the likelihood of false positives. Despite this conservative approach, multiple correlations remain significant, indicating the robustness of our findings.

## 5. Conclusions

Our study provides a detailed analysis of left atrial wall thickness in patients with atrial fibrillation, highlighting the significance of variability within left atrial regions. The anterior wall thickness and left atrial volume index were critical factors associated with AF recurrence post-ablation. Incorporating these wall thickness parameters into clinical practice can enhance understanding atrial structural abnormalities, allowing for more personalised and effective treatment strategies for AF patients.

## Figures and Tables

**Table 1:** Baseline patient characteristics (N = 128).

Baseline Characteristics	
Age (years)	55.6 ± 11.2
BMI (kg/m^2^)	27.5 ± 4.0
LAVI (mL/m^2^)	58.4 ± 17.1
LAV (mL)	132.1 ± 36.4
Ejection Fraction (%)	56.0 ± 9.8
AF type	
Paroxysmal (n, %)	91 (71)
Persistent (n, %)	37 (29)
Female Sex	43 (33.6)
Hypertension	79 (61.7)
Hyperlipidemia	35 (30.8)
Diabetes mellitus	9 (7)
OSAS	16 (12.5)
Smoking	21 (16.4)
COPD	4 (3.1)
Chronic Kidney Disease	4 (3.1)
Coronary Disease	5 (3.9)
Vascular Disease	8 (6.3)
Previous Stroke	3 (2.3)
Thromboembolism	1 (0.8)
Nr comorbidities:	
0	20 (15.6)
1	50 (39.1)
2	35 (27.3)
3	9 (7)
4	6 (4.7)
5	6 (4.7)
6	1 (0.8)
CHA2DS2_VASc Score	
0	26 (20.3)
1	48 (37.5)
2	31 (25)
3	18 (14.1)
4	3 (2.3)
5	1 (0.8)
Recurrence (%)	55 (43)
Follow up time	25.8 ± 17.3

Values are presented as the mean ± SD, n (%). AF, atrial fibrillation; LA, left atrium; OSAS, obstructive sleep apnoea syndrome; COPD, chronic obstructive pulmonary disease; BMI, body mass index; LAV, left atrial volume; LAVI, left atrial volume index.

**Table 2:** Left atrial wall thickness distribution according to the segment. Legend: Segments 1–4, superior wall; 5–6, posterior wall; 7, septal wall; 8–11, anterior wall; 12, left lateral wall; and 13–14, between superior and inferior homolateral pulmonary veins.

		Sex	Thickness (mm)	Mass (g)
Superior Wall	Segment 1	Female	1.289 ± 0.254	0.573 ± 0.214
		Male	1.377 ± 0.678	0.679 ± 0.281
	Segment 2	Female	1.560 ± 0.426	0.649 ± 0.215
		Male	1.708 ± 0.851	0.789 ± 0.292
	Segment 3	Female	1.320 ± 0.280	0.603 ± 0.248
		Male	1.308 ± 0.666	0.736 ± 0.274
	Segment 4	Female	1.354 ± 0.301	0.537 ± 0.188
		Male	1.361 ± 0.692	0.670 ± 0.290
Posterior Wall	Segment 5	Female	1.349 ± 0.283	1.293 ± 0.537
		Male	1.357 ± 0.682	1.428 ± 0.541
	Segment 6	Female	1.382 ±0.284	1.804 ± 0.568
		Male	1.414 ± 0.703	2.088 ± 0.719
Septal Wall	Segment 7	Female	1.245 ± 0.268	1.733 ± 0.567
		Male	1.364 ± 0.678	2.045 ± 0.824
Anterior Wall	Segment 8	Female	1.739 ± 0.562	2.452 ± 0.825
		Male	1.983 ± 0.993	2.806 ± 0.956
	Segment 9	Female	1.256 ± 0.265	0.482 ± 0.152
		Male	1.286 ± 0.651	0.546 ± 0.197
	Segment 10	Female	1.978 ± 0.707	1.510 ± 0.518
		Male	2.226 ± 1.131	1.788 ± 0.547
	Segment 11	Female	1.337 ± 0.291	0.378 ± 0.159
		Male	1.338 ± 0.680	0.438 ± 0.201
Left Lateral Wall	Segment 12	Female	1.304 ± 0.328	0.815 ± 0.391
		Male	1.351 ± 0.701	0.818 ± 0.397
Between Superior Veins	Segment 13	Female	1.805 ± 0.647	0.189 ± 0.117
		Male	1.804 ± 1.004	0.298 ± 0.172
Between Inferior Veins	Segment 14	Female	1.469 ± 0.355	0.423 ± 0.187
		Male	1.590 ± 0.786	0.548 ± 0.322

## Data Availability

Data are available upon reasonable request from the corresponding author.
